# Impact of combined surgery on reoperation rates in odontogenic sinusitis: a retrospective comparative study

**DOI:** 10.1007/s00405-025-09568-6

**Published:** 2025-07-12

**Authors:** Santiago Almanzo, Saúl Astray-Gómez, Inés Tortajada-Torralba, Javier Cabrera-Guijo, Lucas Fito-Martorell, Noelia Muñoz-Fernández, Miguel Armengot-Carceller, Alfonso García-Piñero

**Affiliations:** 1 Department of Otolaryngology, Hospital Universitari i Politècnic La Fe, Avinguda Fernando Abril Martorell 106, Valencia, 46026 Valencia Spain; 2 Department of Surgery, Faculty of Medicine and Dentistry, University of Valencia, Valencia, Spain

**Keywords:** Chronic maxillary sinusitis, Odontogenic sinusitis, Odontogenic maxillary sinusitis, Combined surgical treatment, Endoscopic sinus surgery, Oroantral fistula management

## Abstract

**Purpose:**

Odontogenic sinusitis (ODS) is a frequent but underdiagnosed condition requiring multidisciplinary management. This study aims to compare outcomes between single-stage combined surgical treatment by otolaryngology and maxillofacial surgery teams versus non-combined approaches, and to evaluate whether the type of prior treatment influences the need for reintervention or secondary procedures.

**Methods:**

We conducted a retrospective observational study including 96 patients with ODS surgically treated between January 2019 and December 2024. Patients were categorized according to etiology and surgical strategy (combined vs. non-combined). Additionally, we analyzed whether the type of previous treatment (none, otolaryngology alone, maxillofacial surgery, dentist, or combined) was associated with the need for reoperation or second-stage maxillofacial surgery.

**Results:**

Combined surgery significantly reduced reoperation rates (*p* = 0.003), particularly in periodontic/endodontic infection cases (*p* = 0.002). No significant differences were observed in the need for second-stage maxillofacial surgery between groups. Reoperation was more frequent among patients initially treated by otolaryngology or maxillofacial surgeons, while those managed first by dentists had significantly lower reoperation rates (*p* < 0.001). The type of previous treatment was not significantly associated with second-stage maxillofacial surgery. Etiology and patient age did not influence surgical outcomes.

**Conclusion:**

Single-stage combined surgery reduces the need for reoperation in ODS. When a staged approach is selected, prioritizing initial dental or maxillofacial management before otolaryngologic intervention appears to yield better outcomes.

## Introduction

Odontogenic sinusitis (ODS) is an underdiagnosed condition that accounts for up to 40% of chronic maxillary sinusitis cases [1, 2]. Unlike primary nasal rhinosinusitis, ODS arises from dental infections, iatrogenic dental procedures, or the persistence of an oroantral communication, typically presenting as a unilateral and chronic disease [3].

The symptoms of ODS can overlap with those of conventional maxillary sinusitis, including nasal obstruction, anterior or posterior rhinorrhea, hyposmia, and unilateral facial headache [4]. However, additional symptoms suggestive of a dental origin, such as tooth pain or sensitivity, halitosis, purulent drainage through an oroantral fistula, or cacosmia, are frequently observed [1].

The diagnosis of ODS can be challenging, as its clinical and radiological manifestations may resemble those of rhinogenic sinusitis. However, the identification of unilateral maxillary sinus opacification on computed tomography (CT), along with endoscopic findings such as purulent secretion in the middle meatus, in a patient without a previous rhinitis and the presence of an underlying dental pathology, is key to its diagnosis [5]. A significant percentage of these cases are initially overlooked, leading to therapeutic failures and recurrences [6].

The treatment of ODS requires a multidisciplinary approach [7]. The combination of antibiotics and dental treatment may be sufficient in selected cases, particularly when the dental pathology is mild and there is no significant sinus involvement. However, numerous studies have demonstrated that functional endoscopic sinus surgery (FESS) combined with dental surgery in a single-stage procedure provides superior success rates, reducing complications and recurrences [8, 9]. Despite this, controversies persist regarding whether combined surgery is always necessary or if a staged approach may offer comparable outcomes in terms of disease resolution and recurrence prevention [10].

Recent studies have emphasized the importance of a well-defined treatment protocol to optimize outcomes in ODS cases. Saibene et al. proposed a classification-based treatment strategy that integrates transnasal and intraoral approaches depending on the complexity of the pathology, with promising results [10]. Similarly, Kende et al. demonstrated that a combined endoscopic and intraoral approach significantly improved postoperative quality of life and reduced the risk of persistent sinus disease [11]. These findings reinforce the necessity of individualized patient management strategies based on disease severity and etiology.

In this study, we analyze the outcomes of patients surgically treated for ODS, comparing two therapeutic strategies: single-stage combined otolaryngologic and dental surgery versus a non-combined strategy. We also explore whether the type of prior treatment— performed by an otolaryngologist, an oral and maxillofacial surgeon, or dental professionals—has an impact on surgical outcomes. Our objective is to determine whether combined surgery is essential for disease resolution or if an alternative approach can provide comparable results in terms of disease control and recurrence.

## Materials and methods

A single-center observational study was conducted at a tertiary hospital, including patients diagnosed with ODS who underwent surgical treatment between January 2019 and December 2024. Surgical recruitment concluded in December 2024, and all patients were followed for a minimum of six months postoperatively. The final patient’s follow-up was completed in June 2025. Diagnosis was established based on nasal endoscopy, evidencing unilateral purulence in the middle meatus, and paranasal sinus CT, which demonstrated maxillary sinus opacification with adjacent dental pathology.

To ensure sample homogeneity, patients were classified according to the underlying cause of ODS into three categories: (a) Periodontic/endodontic infection, which included cases of periodontitis, apical periodontitis, chronic periapical lesions, failed endodontic treatments, and periapical cysts resulting in secondary maxillary sinusitis; (b) Oroantral communication, secondary to previous tooth extraction, Caldwell-Luc surgery, osteoradionecrosis, bone dehiscence, or impacted third molars; and (c) Implant-related ODS, either due to peri-implant infection or displacement of the implant into the maxillary sinus. All cases were identified through a retrospective review of diagnostic and surgical codes and were consecutively included if they had a confirmed diagnosis of ODS based on clinical, radiologic, and/or intraoperative findings. No specific etiologic subtypes were excluded.

Patients underwent surgical intervention according to two treatment strategies. The combined surgery group included cases where both the Otolaryngology – Head and Neck Surgery (OHNS) and Oral and Maxillofacial Surgery (OMFS) teams performed a joint procedure in a single operative session. OHNS procedures consisted of FESS aimed at ventilating and draining the maxillary sinus and addressing any associated inflammation. Maxillofacial procedures included dental extractions, apicoectomies, periapical cyst removal, implant removal, or closure of oroantral fistulas.

The non-combined group included patients who underwent surgery exclusively by the OHNS team, without simultaneous OMFS participation. In some of these cases, patients had previously undergone maxillofacial or dental procedures in a separate surgical act or clinical setting.

Treatment allocation was determined based on interdisciplinary assessment and patient-specific characteristics. More severe or anatomically complex cases were more likely to be managed with a combined approach. We acknowledge that this may introduce a selection bias, which is addressed in the Discussion.

In addition to clinical and demographic variables, we analyzed two key postoperative outcomes: (1) Reoperation, defined as any additional surgical procedure performed by the OHNS team—either alone or in conjunction with OMFS—due to persistent or recurrent symptoms at least one month after the initial surgery; and (2) Second-stage maxillofacial surgery, defined as a subsequent surgical procedure performed exclusively by the OMFS team, typically in cases where the dental pathology was not addressed during the first intervention. The primary comparison was conducted between patients treated with combined surgery and those who underwent a non-combined surgical approach. In addition, we explored whether the type of prior treatment—performed by an OHNS, an OMFS, or dental professionals—had any association with the need for reoperation or second-stage maxillofacial surgery, regardless of the surgical strategy employed during the study. For this analysis, “previous dental treatment” included procedures such as endodontic therapy, dental extractions, or implant removal performed by general dentists.

The study was approved by the Ethics Committee of the hospital, and all procedures were performed in accordance with the ethical standards of the Declaration of Helsinki. Statistical analysis was performed using Jamovi software (version 2.6.44). Chi-square or Fisher’s exact tests were used to compare categorical variables, with the latter applied in cases of small sample sizes (i.e., expected cell count < 5). Continuous variables, such as age, were compared using Student’s t-test, assuming normal distribution based on inspection of means and ranges. A p-value < 0.05 was considered statistically significant.

## Results

A total of 96 patients diagnosed with ODS were included, with a mean age of 52.8 ± 13.7 years. Of these, 59 (61.5%) were male and 37 (38.5%) were female. The most frequent etiologies were periodontic/endodontic infection and oroantral communication, each present in 42 patients (43.8%), followed by implant-related ODS in 12 patients (12.5%).

Regarding surgical strategy, in the periodontic/endodontic infection group, 18 patients (42.9%) underwent combined surgery (OHNS + OMFS), while 24 (57.1%) were treated with non-combined surgery by OHNS alone. In the oroantral communication group, 23 patients (54.8%) received combined surgery, while 19 (45.2%) underwent non-combined surgery. In the implant-related ODS group, 3 patients (25%) underwent combined surgery, and 9 (75%) underwent non-combined surgery.

The need for second-stage maxillofacial surgery did not show significant differences between the combined and non-combined surgery groups (*p* = 0.060). In the periodontic/endodontic infection group, second-stage maxillofacial surgery was required in 1 patient (5.6%) with combined surgery and in 6 patients (25%) with non-combined surgery (*p* = 0.208). In the oroantral communication group, second-stage surgery was needed in 3 patients (13%) with combined surgery and in 4 patients (21.1%) with non-combined surgery (*p* = 0.682). In the implant-related ODS group, second-stage maxillofacial surgery was required in none of the patients who underwent combined surgery, and in 3 patients (33.3%) who underwent non-combined surgery (*p* = 0.509).

**Fig. 1:**
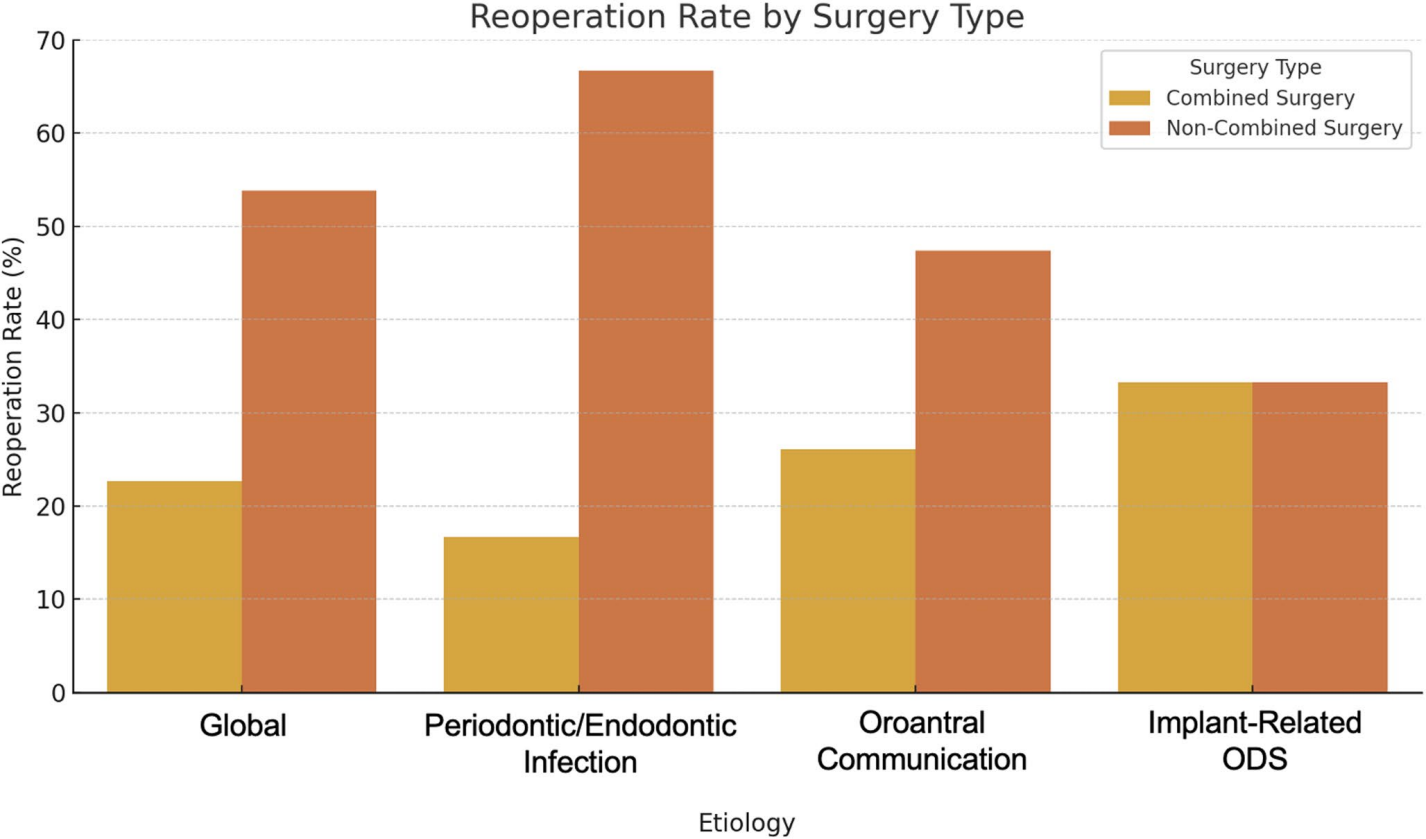
Reoperation rate by etiology and surgical strategy. Overall, the reoperation rate was significantly lower in the combined surgery group compared to the non-combined surgery group (22.7% vs. 53.8%, *p* = 0.003). In the periodontic/endodontic infection subgroup, this difference was also statistically significant (16.7% vs. 66.7%, *p* = 0.002). In the oroantral communication group, although the difference between combined and non-combined surgery (26.1% vs. 47.4%) was notable, it did not reach statistical significance (*p* = 0.202). No differences were observed in the implant-related ODS group (33.3% vs. 33.3%, *p* = 1.0)

Regarding the need for reoperation due to persistent symptoms or complications, a higher frequency was observed in patients who underwent non-combined surgery (Fig. 1). Overall, combined surgery significantly reduced reoperation rates (*p* = 0.003).

When analyzing the different etiological groups, in the periodontic/endodontic infection group, 16.7% (3/18) of patients treated with combined surgery required a reoperation, whereas this rate increased to 66.7% (16/24) in the non-combined surgery group, showing a statistically significant difference (*p* = 0.002). In the oroantral communication group, reoperation was necessary in 6 patients (26.1%) with combined surgery and in 9 patients (47.4%) with non-combined surgery (*p* = 0.202). In the implant-related ODS group, reoperation was required in 1 patient (33.3%) with combined surgery and in 3 patients (33.3%) with non-combined surgery (*p* = 1.0).

When comparing the reoperation rate among the three ODS groups, regardless of the type of surgery performed, no significant differences were found (*p* = 0.600). Similarly, the comparison of second-stage maxillofacial surgery among the ODS etiological groups did not show statistically significant differences (*p* = 0.779). Additionally, no significant differences were observed in the mean age of patients regarding the need for reoperation (*p* = 0.669) or second-stage maxillofacial surgery (*p* = 0.706).

**Fig. 2:**
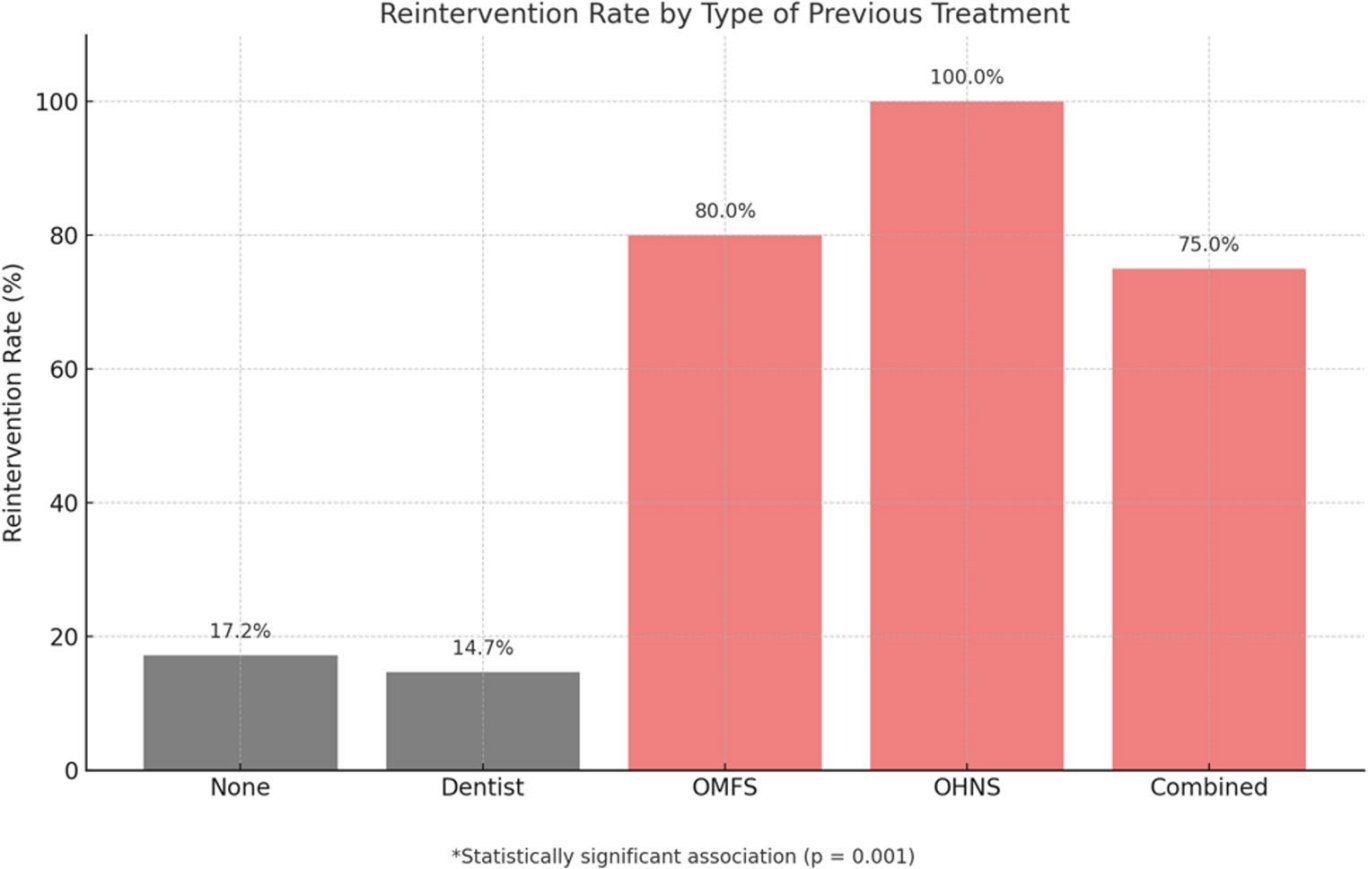
Reintervention rate by type of previous treatment. Patients who had received prior treatment exclusively by OHNS or OMFS showed the highest reoperation rates (100% and 80%, respectively). In contrast, those initially treated by a dentist had a significantly lower reoperation rate (14.7%). The overall association between previous treatment type and the need for reoperation was statistically significant (*p* < 0.001)

Analysis of outcomes based on previous treatment approach revealed a statistically significant association between the type of initial intervention and the need for reoperation (*p* < 0.001). Patients who had received prior treatment by OHNS alone (FESS group) or by OMFS exhibited higher reoperation rates (9/9 and 16/20, respectively), while those initially managed by a dentist showed a significantly lower rate (5/34). These results are illustrated in Fig. 2.

In contrast, no significant association was found between the type of previous treatment and the need for second-stage maxillofacial surgery (*p* = 0.258). Among patients treated previously by a dentist or maxillofacial surgeon, the majority did not require additional OMFS intervention (30/34 and 17/20 respectively), suggesting that initial dental or OMFS management may contribute to a lower need for subsequent maxillofacial surgery, although without reaching statistical significance.

## Discussion

Odontogenic sinusitis has a higher prevalence than historically reported, accounting for up to 40% of chronic maxillary sinusitis cases [2, 3]. In our series, all analyzed patients had a confirmed diagnosis of ODS, highlighting the relevance of this pathology in otolaryngology practice.

The optimal treatment strategy for ODS remains a topic of debate, particularly regarding the role of a multidisciplinary surgical approach. Kocum et al. (2024) found that single-stage combined surgery involving both OHNS and OMFS achieved a 97% success rate, preventing reoperations and complications [8]. In our study, combined surgery significantly reduced the need for reoperation compared to non-combined surgery (*p* = 0.003), reinforcing its role in optimizing surgical outcomes. However, it is important to acknowledge that the choice between combined and non-combined strategies was not based on a standardized protocol, but rather on clinical criteria and the availability of surgical teams. Consequently, patients undergoing combined surgery may have presented with more complex clinical scenarios, potentially introducing selection bias. This factor limits the ability to draw causal inferences from the observed associations. Based on our experience and existing evidence, early involvement of OMFS may be particularly beneficial in cases with complex dental pathology, such as advanced periodontal disease, periapical cysts, oroantral fistulas, or displaced implants. A multidisciplinary surgical approach in these scenarios may optimize outcomes by addressing both the sinonasal and dental components of the disease simultaneously.

When analyzing the different etiological subgroups, we observed that the reduction in reoperation rate with combined surgery reached statistical significance only in patients with periodontic/endodontic infection (*p* = 0.002). In contrast, the differences between combined and non-combined approaches were not statistically significant in the oroantral communication (*p* = 0.202) and implant-related ODS groups (*p* = 1.0). Notably, the implant subgroup included only 12 patients, which limits the statistical power to detect meaningful differences. This is particularly relevant given that prior literature has suggested a more complex clinical course in implant-associated cases, with a higher risk of recurrence [1, 6]. Therefore, although our findings did not replicate this trend, we believe the results should be interpreted with caution and not taken as definitive. Larger prospective studies are needed to better characterize the impact of surgical strategy in these specific etiologies.

Another relevant aspect is that combined surgery did not significantly impact the need for second-stage maxillofacial surgery. Craig et al. (2022) suggested that some patients may benefit from a sequential surgical approach rather than an initial combined intervention [5]. Our data reinforce this possibility, as the need for additional maxillofacial surgery showed no significant differences between the combined and non-combined groups. As a retrospective study, we recognize that combined surgery may have been favored in more severe or persistent cases, potentially introducing a treatment selection bias.

When comparing postoperative evolution across the three etiological groups, our results showed no significant differences in reoperation rates (*p* = 0.600) or in the need for additional surgery (*p* = 0.779). This suggests that the etiology of ODS alone is not a determinant factor in postoperative prognosis. Felisati et al. (2013) similarly emphasized that surgical success is primarily dependent on selecting the appropriate treatment strategy and effectively addressing the underlying dental pathology rather than on the specific etiological classification [7]. In contrast, some studies have reported worse outcomes in cases related to implants or long-standing oroantral fistulas [1, 6].

Our analysis of previous treatments revealed that patients initially treated by OHNS had significantly higher reoperation rates compared to those who received initial dental or maxillofacial treatment, regardless of whether the subsequent surgery was combined or non-combined (*p* < 0.001). Additionally, the need for second-stage maxillofacial surgery was more frequent among patients initially treated by OHNS than those managed by dental or OMFS professionals, although this difference did not reach statistical significance (*p* = 0.145). These findings support the potential benefit of prioritizing dental or maxillofacial intervention in a staged surgical approach, addressing the dental pathology before OHNS intervention.

Furthermore, patient age did not influence postoperative outcomes. No significant differences were observed between patients who required reoperation and those who did not (*p* = 0.669), nor between those who required second-stage maxillofacial surgery and those who did not (*p* = 0.706). This finding aligns with previous studies showing that age is not a key determinant in the success of surgical treatment for ODS [4].

To ensure reliability of outcome assessment, all patients had a minimum follow-up period of six months after surgery. The final patient included was operated on in December 2024, and follow-up data were reviewed and completed by June 2025.

These findings should be interpreted considering the limitations of a retrospective, single-center design and limited sample size, which may reduce the generalizability of the results to broader clinical settings.

Recent studies have highlighted that early surgical intervention in symptomatic ODS cases can lead to faster symptom resolution and improved endoscopic outcomes compared to delayed management [11]. However, other authors have proposed a stepwise treatment model, advocating for initial management of the dental pathology followed by endoscopic sinus surgery only if symptoms persist or sinus opacification remains unresolved. In this regard, Craig et al. [12] emphasized the value of addressing the odontogenic source first—particularly when clearly identifiable—and delaying FESS until after dental resolution, especially in cases with mild sinus disease. These contrasting approaches underscore the need for individualized treatment planning based on clinical severity, symptom burden, and radiological findings.

From an economic and healthcare perspective, previous studies suggest that reducing the need for reoperation through combined surgery could result in lower hospital burden, fewer readmissions, and decreased surgical and anesthetic resource utilization [3, 8, 10]. In particular, a single-stage multidisciplinary approach may reduce overall treatment duration and the number of hospital visits, which translates into shorter cumulative hospital stays and lower indirect costs for patients and healthcare systems. Previous studies have demonstrated that minimizing reinterventions leads to a reduction in overall healthcare costs and improves hospital efficiency, reinforcing the importance of optimizing ODS management in tertiary care hospitals [13–15].

## Conclusion

Single-stage combined OHNS-OMFS surgery was associated with a reduction in the need for reoperation, particularly in the periodontic/endodontic infection subgroup However, no significant impact was observed regarding the need for second-stage maxillofacial surgery, suggesting that a staged surgical approach may be a valid alternative in some cases. Based on our findings, if a staged surgical approach is chosen, initiating treatment with dental or maxillofacial management before OHNS intervention appears to yield better outcomes.

The etiology of ODS, whether periodontic/endodontic infection, oroantral communication, or implant-related, did not significantly influence reoperation rates or the need for additional surgical intervention.

## References

[1] Little RE, Long CM, Loehrl TA, Poetker DM (2018) Odontogenic sinusitis: a review of the current literature. Laryngoscope Investig Otolaryngol 3(2):110-117. 10.1002/lio2.147 10.1002/lio2.147 PMC5915825 29721543

[2] Lechien JR, Filleul O, de Araujo PC, Hsieh JW, Chantrain G, Saussez S (2014) Chronic maxillary rhinosinusitis of dental origin: a systematic review of 674 patient cases. Int J Otolaryngol 2014:465173. 10.1155/2014/465173 10.1155/2014/465173 PMC4000986 24817890

[3] Craig JR, Tataryn RW, Aghaloo TL, Pokorny AT, Gray ST, Mattos JL et al (2020) Management of odontogenic sinusitis: multidisciplinary consensus statement. Int Forum Allergy Rhinol 10(7):901–912. 10.1002/alr.22598 32506807 10.1002/alr.22598

[4] Psillas G, Papaioannou D, Petsali S, Dimas GG, Constantinidis J (2021) Odontogenic maxillary sinusitis: a comprehensive review. J Dent Sci 16(1):474–481. 10.1016/j.jds.2020.08.001 33384837 10.1016/j.jds.2020.08.001 PMC7770314

[5] Craig JR (2022) Odontogenic sinusitis: a state-of-the-art review. World J Otorhinolaryngol Head Neck Surg 8(1):8–15. 10.1002/wjo2.9 35619928 10.1002/wjo2.9 PMC9126162

[6] Aukštakalnis R, Simonavičiūtė R, Simuntis R (2018) Treatment options for odontogenic maxillary sinusitis: a review. Stomatologija 20(1):22–26 29806655

[7] Felisati G, Chiapasco M, Lozza P et al (2013) Sinonasal complications resulting from dental treatment: outcome-oriented proposal of classification and surgical protocol. Am J Rhinol Allergy 27(4):e101–e106. 10.2500/ajra.2013.27.3936 23883801 10.2500/ajra.2013.27.3936

[8] Kocum P, Sedý J, Traboulsi J, Jirák P (2024) One-stage combined ENT and dental surgical treatment of odontogenic sinusitis: a prospective study. Eur Arch Otorhinolaryngol 281(4):1347–1356. 10.1007/s00405-023-08332-y 37982839 10.1007/s00405-023-08332-y PMC10858141

[9] Stammberger H, Posawetz W (1990) Functional endoscopic sinus surgery: concept, indications, and results of the Messerklinger technique. Eur Arch Otorhinolaryngol 247(2):63–76. 10.1007/BF00183169 10.1007/BF00183169 2180446

[10] Saibene AM, Collurà F, Pipolo C, Bulfamante AM, Lozza P et al (2019) Odontogenic rhinosinusitis and sinonasal complications of dental disease or treatment: prospective validation of a classification and treatment protocol. Eur Arch Otorhinolaryngol 276(2):401–406. 10.1007/s00405-018-5220-0 30483941 10.1007/s00405-018-5220-0 PMC6394426

[11] Kende P, Mathai PC, Landge J, Aggarwal N, Ghodke M et al (2019) Combined endoscopic and intra-oral approach for chronic maxillary sinusitis of dental origin-a prospective clinical study. Oral Maxillofac Surg 23(4):429–437. 10.1007/s10006-019-00792-z 31332583 10.1007/s10006-019-00792-z

[12] Craig JR, McHugh CI, Griggs ZH, Peterson EI (2019) Optimal timing of endoscopic sinus surgery for odontogenic sinusitis. Laryngoscope 129(9):1976–1983. 10.1002/lary.28001 31012972 10.1002/lary.28001

[13] Healy MA, Mullard AJ, Campbell DA Jr, Dimick JB (2016) Hospital and payer costs associated with surgical complications. JAMA Surg 151(9):823–830. 10.1001/jamasurg.2016.0773 27168356 10.1001/jamasurg.2016.0773

[14] Chagpar AB, Killelea BK, Tsangaris TN, Butler M, Stavris K, Li F et al (2015) A randomized, controlled trial of cavity shave margins in breast Cancer. N Engl J Med 373(6):503–510. 10.1056/NEJMoa1504473 26028131 10.1056/NEJMoa1504473 PMC5584380

[15] McCahill LE, Single RM, Aiello Bowles EJ, Feigelson HS, James TA, Barney T et al (2012) Variability in reexcision following breast conservation surgery. JAMA 307(5):467–475. 10.1001/jama.2012.43 22298678 10.1001/jama.2012.43

